# Development and Validation of a CNN-Based Diagnostic Pipeline for the Diagnosis of Otitis Media

**DOI:** 10.3390/jcm14238572

**Published:** 2025-12-03

**Authors:** Hee Won Seo, Dong Woo Ko, Jaehoon Oh, Juncheol Lee, Yong Bae Ji, Sang-Yoon Han, Byeong In Moon, Jae Hoon Jeong, Jae Ho Chung

**Affiliations:** 1 Department of Otolaryngology, College of Medicine, Hanyang University, Seoul 04763, Republic of Korea; heewon31seo@hanyang.ac.kr (H.W.S.); jyb20000@hanyang.ac.kr (Y.B.J.); hsy9010@hanyang.ac.kr (S.-Y.H.); 2 AIDOT Inc., Seoul 05854, Republic of Korea; koodoowoo@aidot.ai (D.W.K.); bluein@aidot.ai (B.I.M.); jman@aidot.ai (J.H.J.); 3 Department of Emergency Medicine, College of Medicine, Hanyang University, Seoul 04763, Republic of Korea; ojjai@hanyang.ac.kr (J.O.); doldoly@hanyang.ac.kr (J.L.)

**Keywords:** tympanic membrane, ear drum, otoscopic image, artificial intelligence, diagnosis, otitis media, acute otitis media, chronic otitis media, otitis media with effusion

## Abstract

**Background/Objectives:** Accurate diagnosis of otitis media (OM) using otoscopic images is often challenging, particularly for non-specialists. Artificial intelligence (AI), especially deep learning-based methods, has shown promising results in supporting the classification of tympanic membrane conditions. This study aimed to develop and validate a multi-step CNN-based AI diagnostic pipeline for the automated classification of tympanic membrane images into four OM categories: normal, acute otitis media (AOM), otitis media with effusion (OME), and chronic otitis media (COM). **Methods:** A total of 2964 otoscopic images were retrospectively collected and annotated by expert otologists. The proposed pipeline consisted of four sequential stages: image quality assessment, tympanic membrane segmentation, side (left/right) classification, and final disease classification. CNN-based deep learning models including MambaOut, CaraNet, EfficientNet, and ConvNeXt were employed in each stage. **Results:** The image quality classifier achieved an accuracy of 98.8%, while the laterality classifier reached 99.1%. For disease classification, the ConvNeXt model demonstrated an overall accuracy of 88.7%, with disease-specific F1-scores of 0.78 for AOM, 0.87 for OME, and 0.92 for COM. The system performed reliably across all stages, indicating strong potential for clinical application. **Conclusions:** The proposed AI pipeline enables automated and accurate classification of tympanic membrane images into common OM subtypes. Its integration into digital otoscopes could support more consistent diagnosis in primary care and underserved settings, while also providing educational support for trainees and general practitioners.

## 1. Introduction

Recent advancements in artificial intelligence (AI), especially deep learning techniques, have markedly improved diagnostic accuracy and clinical applicability in otitis media management [1,2,3,4,5,6]. Numerous studies have demonstrated that deep learning-based AI models effectively classify various types of middle ear diseases, such as acute otitis media (AOM), otitis media with effusion (OME), chronic otitis media (COM), and cholesteatoma, based on tympanic membrane images [1,4,5,7,8,9]. A recent meta-analysis reported accuracies ranging from approximately 70% to 97%, highlighting AI’s potential as a reliable diagnostic aid in clinical practice [10].

In routine clinical settings, the initial diagnosis of otitis media typically relies on visual inspection of the tympanic membrane [11]. However, diagnostic accuracy varies significantly depending on examiner expertise. Even specialists in otolaryngology achieve a diagnostic accuracy of around 70%, whereas general practitioners and pediatricians demonstrate considerably lower accuracy [10]. Thus, AI-assisted automated diagnostic systems have the potential to enhance diagnostic precision among general physicians, promote early detection, and encourage timely therapeutic interventions, ultimately improving patient outcomes.

For real-world clinical use, it is beneficial to integrate AI technology into digital otoscopes. Digital otoscopes can provide high-resolution tympanic membrane images and real-time AI-driven diagnostic insights, significantly enhancing the efficiency of clinical evaluations. However, for successful clinical implementation, the AI system must automatically assess whether captured tympanic membrane images are diagnostically acceptable. Furthermore, it is essential to accurately segment the region of interest (ROI)—specifically the tympanic membrane—prior to diagnostic classification. This preprocessing step helps the AI model exclude irrelevant regions such as the external auditory canal, enabling it to focus specifically on the pathological features associated with OM, thereby further improving diagnostic accuracy.

The aim of the present study was to develop an artificial intelligence diagnostic system suitable for clinical integration into digital otoscopes. The system will classify tympanic membrane images into categories including normal, acute otitis media (AOM), chronic otitis media (COM), and otitis media with effusion (OME), thus facilitating precise and efficient diagnosis.

## 2. Materials and Methods

### 2.1. Tympanic Membrane Data Acquisition and Annotation

The current study conducted a retrospective review of medical records and otoscopic images collected from patients who visited the otolaryngology outpatient clinic for ear-related symptoms between January 2020 and December 2022. Tympanic membrane images were acquired using a digital videoscope (ENF-V2, Olympus, Tokyo, Japan) and archived in the institution’s Picture Archiving and Communication System (PACS). All images were extracted in JPEG format with a resolution of 640 × 480 pixels for subsequent analysis. Each image was annotated with a clinically confirmed diagnosis by a senior otology specialist based on visual otoscopic findings. Diagnoses were categorized into four groups: normal tympanic membrane, acute otitis media (AOM), otitis media with effusion (OME), and chronic otitis media (COM). AOM was defined by a bulging and erythematous tympanic membrane suggestive of acute inflammation with purulent effusion; OME was identified by a translucent or retracted membrane, often accompanied by air–fluid levels or bubbles without signs of acute infection; and COM included tympanic membranes with chronic structural changes such as perforations, tympanosclerosis, or retraction pockets, frequently associated with persistent or recurrent otorrhea. A total of 2964 otoscopic images were included in the dataset, consisting of 1052 normal cases, 232 AOM cases, 700 OME cases, and 980 COM cases.

### 2.2. AI Diagnostic Pipeline

The present study developed an AI-based diagnostic pipeline named ECHO-dot-AI (model: ADT-ES10, AIDOT Inc., Seoul, Republic of Korea) comprising four sequential modules—image quality classification, tympanic membrane segmentation, laterality determination, and disease classification—mirroring clinical reasoning in otitis media diagnosis.

**Figure 1:**
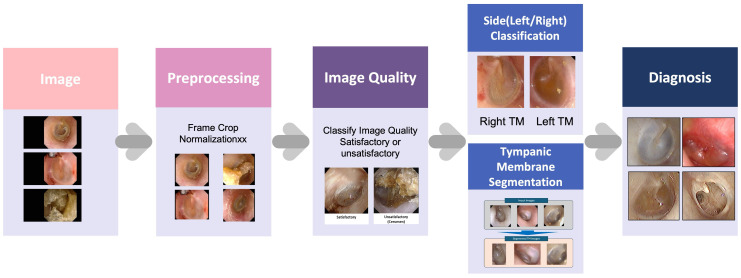
Overview of the artificial intelligence-based otoscopic diagnostic pipeline for otitis media.

Specifically, the image quality module uses MambaOut, a lightweight image quality classifier that filters out blurred, poorly illuminated, or improperly focused otoscopic images so that only diagnostically reliable photographs are analyzed. The segmentation module is implemented with CaraNet, a network optimized for small medical structures that automatically delineates the tympanic membrane and removes surrounding ear canal structures, allowing the system to focus on the clinically relevant region. For laterality determination, we adopted EfficientNet-B0, a compact convolutional neural network (CNN) that recognizes anatomical landmarks such as the malleus to decide whether the image shows a right or left eardrum. Finally, disease classification is performed by ConvNeXt, a modern CNN that assigns each image to one of four diagnostic categories (normal, acute otitis media, otitis media with effusion, and chronic otitis media). Each module incorporates a task-optimized deep learning model selected for its efficiency, accuracy, and suitability for clinical application (Figure 1).

#### 2.2.1. Image Preprocessing and Data Augmentation

Prior to model training, otoscopic images were cropped using a rule-based algorithm to remove non-diagnostic peripheral padding. Images were normalized using dataset-specific means and standard deviations and resized per model requirements. Augmentation techniques—including horizontal/vertical flips (except for laterality), ±15° rotation, brightness/contrast variation, and random cropping—were applied to simulate clinical variability and improve model generalization.

#### 2.2.2. Image Quality Classification

To filter out low-quality images, the present study employed the MambaOut model for binary classification of diagnostic image quality [12]. MambaOut, which replaces the state–space model (SSM) token mixer in the original Mamba architecture with Gated CNN blocks, offers a lightweight, efficient model that achieves competitive accuracy in image classification while operating with significantly fewer parameters and FLOPs. The model’s architecture enables robust inference under clinically variable lighting and focus conditions.

#### 2.2.3. Tympanic Membrane Segmentation

To isolate the tympanic membrane area, image segmentation was performed using CaraNet [13], which employs context axial reverse attention to enhance the segmentation of small anatomical structures. CaraNet includes channel-wise feature pyramid modules and axial reverse attention mechanisms to preserve fine boundary delineation in challenging images. Its architecture provides accurate tympanic membrane image extraction with computational efficiency, essential for the downstream diagnostic stages.

#### 2.2.4. Laterality Classification

To distinguish between left and right tympanic membrane images—a critical factor since middle ear conditions can differ between ears—we utilized EfficientNet-B0 for determining the laterality (left vs. right) of the tympanic membrane by leveraging the malleus orientation [14]. EfficientNet was chosen due to its compound scaling approach, balancing depth, width, and resolution effectively to deliver high accuracy with minimal model size, enabling swift and reliable classification suitable for deployment.

#### 2.2.5. Disease Classification

**Figure 2:**
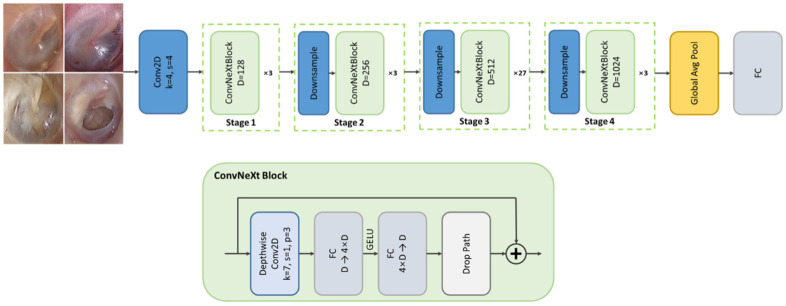
Architecture of the ConvNeXt-based otitis media classification model.

The final stage of the diagnostic pipeline performed multi-class classification of otoscopic images into four clinically meaningful categories—normal, acute otitis media (AOM), otitis media with effusion (OME), and chronic otitis media (COM)—by leveraging the ConvNeXt architecture, a state-of-the-art convolutional neural network that incorporates design principles inspired by vision transformers while maintaining the computational efficiency and inductive bias of traditional CNNs [15]. ConvNeXt was selected for its superior performance on large-scale image recognition benchmarks and its robustness in handling subtle variations in visual patterns, such as color, texture, and contour differences frequently observed across middle ear disease types. Its architectural features—such as large kernel convolutions (7 × 7), layer normalization, and GELU activations—enable improved representational capacity and stable training dynamics, which are essential when distinguishing between diagnostically similar conditions under variable clinical imaging environments. The model’s hierarchical feature extraction structure is particularly well-suited to capturing fine-grained pathological cues present in the tympanic membrane. To minimize anatomical inconsistencies and labeling noise, only otoscopic images that had passed quality assessment, been accurately segmented, and correctly labeled for side (left/right) orientation were used as input. This careful preselection, coupled with ConvNeXt’s strong generalization ability, allowed the system to generate reliable diagnostic outputs suitable for clinical decision support (Figure 2).

### 2.3. Training and Evaluation

All models were implemented in PyTorch v2.9.0 and trained on NVIDIA RTX 4090 GPUs. Three optimizers—Adam, AdamW, and Lion—were tested for classification and segmentation tasks, and AdamW was chosen for all modules due to its better training stability and generalization [16,17,18]. Loss functions were task-specific: cross-entropy loss for classification modules and combined binary cross-entropy with weighted IoU for segmentation [19]. Performance was evaluated using standard metrics: accuracy, sensitivity, specificity, precision, and F1-score for classification, and mean Dice coefficient and IoU for segmentation. In addition, this study used five-fold cross-validation to evaluate the generalization performance of the disease classification model, and for each fold, we calculated receiver operating characteristic (ROC) curves and the corresponding area under the curve (AUC) to assess discriminative ability.

### 2.4. Ethics

This study was approved by the Institutional Review Board (IRB) of Hanyang University Hospital (IRB No. HYUH 2024-12-029) and conducted in accordance with the principles of the Declaration of Helsinki. All personal identifiers were removed during data collection, and the requirement for informed consent was waived due to the retrospective nature of the study.

## 3. Results

The proposed AI diagnostic pipeline was evaluated through a series of five-fold cross-validation experiments using otoscopic images categorized into normal, AOM, OME, and COM. Each component of the pipeline was assessed separately for performance.

### 3.1. Satisfactory vs. Unsatisfactory Image Classification

**Figure 3:**
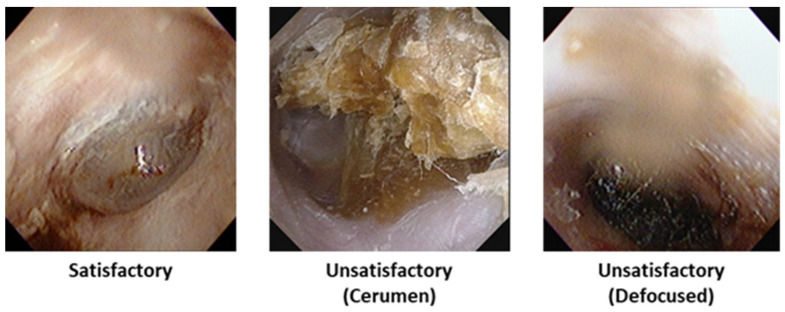
Examples of otoscopic images categorized based on diagnostic usability, according to image quality.

The first stage of the pipeline focused on determining whether an image was suitable for diagnostic analysis. Images were categorized as either ‘satisfactory’ or ‘unsatisfactory’ depending on the visibility of the tympanic membrane. Images obscured by cerumen or degraded by poor focus or motion blur were excluded from downstream analysis. The MambaOut model was used for this classification and achieved an overall accuracy of 98.81%. The classification accuracy for satisfactory images was 93.02%, and for unsatisfactory images, the accuracy was 93.75% when due to cerumen and 99.35% when due to other image quality issues (Figure 3).

### 3.2. Results of Tympanic Membrane Segmentation

**Figure 4:**
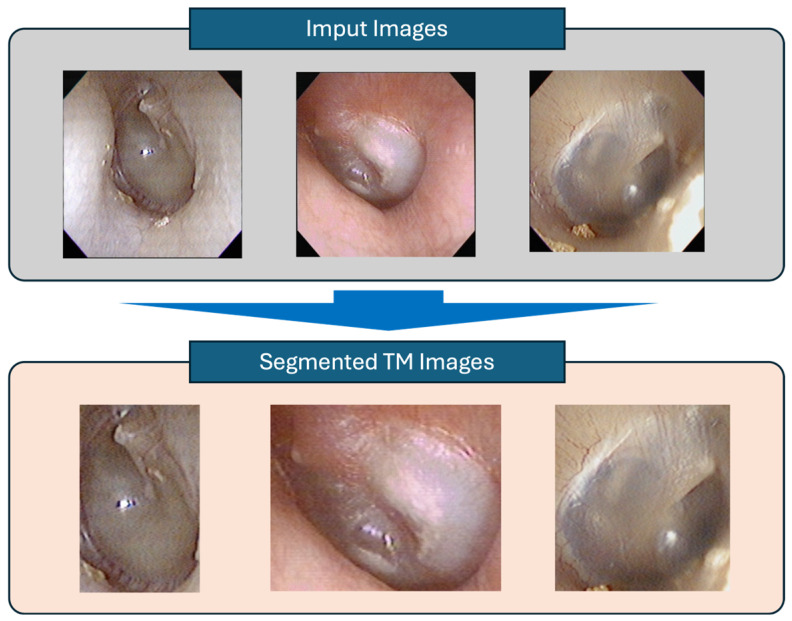
Examples of tympanic membrane regions extracted from otoscopic images by the segmentation model.

To enhance diagnostic performance by removing irrelevant regions (e.g., external auditory canal and dark background), segmentation of the tympanic membrane was performed using the CaraNet model. By isolating the region of interest (ROI), the model learned features specific to otitis media pathology more efficiently. Visual inspection of segmentation outputs confirmed that the model successfully extracted the tympanic membrane area from full otoscopic images. The segmentation model achieved a mean Dice coefficient of 0.94 and a mean Intersection over Union (IoU) of 0.89, indicating high agreement with the ground truth (Figure 4).

### 3.3. Left/Right Tympanic Membrane Classification

To improve anatomical interpretation, the pipeline included a step to classify the laterality (left vs. right) of the tympanic membrane based on the direction of the malleus. This task was carried out using the EfficientNet model, which achieved a classification accuracy of 99.06%.

### 3.4. Otitis Media Classification

The final stage classified the segmented and satisfactory images into four diagnostic categories: normal, AOM, OME, and COM. The ConvNeXt model was used for this multi-class classification task. Classification was performed using the ConvNeXt deep learning model. The model achieved an overall accuracy of 88.66% with a sensitivity of 92.15% and specificity of 91.25% across all categories. The class-wise evaluation based on the confusion matrix showed that the model achieved a precision of 0.86, recall of 0.91, and F1-score of 0.89 for normal tympanic membranes (support: 1052). For acute otitis media (AOM), the precision was 0.79, recall was 0.77, and F1-score was 0.78 (support: 232). Otitis media with effusion (OME) was classified with a precision of 0.90, recall of 0.84, and F1-score of 0.87 (support: 700). Chronic otitis media (COM) achieved a precision of 0.93, recall of 0.92, and F1-score of 0.92 (support: 980) (Table 1 and Table 2).

**Figure 5:**
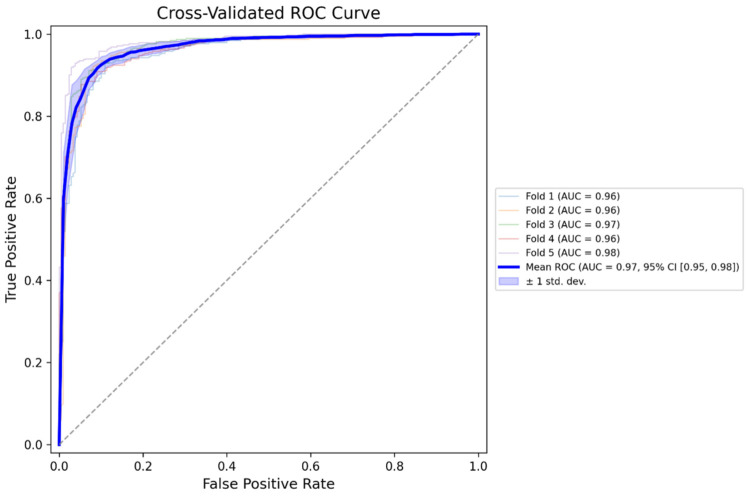
Cross-validated receiver operating characteristic (ROC) curves for the otitis media classification model.

To assess the generalization performance of the model, five-fold cross-validation was conducted. The results showed consistent classification performance, with accuracy ranging from 87.35% to 91.55%, sensitivity from 91.10% to 93.19%, and specificity from 89.10% to 96.19% (Table 3). The ROC curve averaged over the five folds demonstrated a high discriminative ability, with fold-wise AUC values between 0.9584 and 0.9818 (Figure 5).

## 4. Discussion

The present study developed a multi-step deep learning-based diagnostic pipeline to classify tympanic membrane images into four categories of normal, AOM, OME, and COM. The pipeline incorporated four sequential modules: image quality assessment, tympanic membrane segmentation, side (left/right) classification, and final disease classification. The model demonstrated an overall classification accuracy of 88.66% on the external validation dataset (*n* = 2964), with disease-specific F1-scores ranging from 0.78 (AOM) to 0.92 (COM). The segmentation and side classification models also showed high performance, achieving accurate tympanic membrane localization, and left–right discrimination. The model also demonstrated strong discriminative performance, with area under the ROC curve (AUC) values ranging from 0.9584 to 0.9818 across a five-fold cross-validation, indicating a high ability to distinguish between diagnostic categories regardless of data partitioning. These results suggest that the proposed pipeline is capable of processing real-world otoscopic images through multiple stages and delivering automated classification of otitis media subtypes with clinically meaningful performance.

Accurate diagnosis of otitis media (OM) remains a clinical challenge, particularly in settings lacking otolaryngology specialists. Otoscopic interpretation requires the ability to distinguish subtle differences in tympanic membrane appearance, which may be hindered by limited training or clinical exposure. Several studies have highlighted the limitations in diagnostic accuracy among non-specialists, including general practitioners and primary care or pediatric residents. A study evaluated the diagnostic performance of pediatric residents in comparison to pediatric otolaryngologists, using both clinical assessment and tympanometry as reference standards [1,2,3,4,5]. In this analysis involving 79 ear examinations, the sensitivity and specificity of the pediatric residents in differentiating abnormal (acute otitis media or otitis media with effusion) from normal tympanic membranes were 60.9% and 78.8%, respectively. Another study demonstrated that trainees in primary care disciplines had a diagnostic accuracy of only 30% prior to receiving any formal training, highlighting the substantial limitations in otoscopic interpretation among untrained general practitioners [20].

Given the inherent difficulty of accurately diagnosing middle ear diseases through otoscopy, especially in clinical environments without otolaryngology specialists, there is growing interest in the application of AI to support diagnostic decision-making. The interpretation of tympanic membrane findings can be particularly challenging for non-specialists and early-stage trainees, with prior studies reporting limited sensitivity and diagnostic accuracy in such groups. In this context, AI-based diagnostic tools may serve not only as decision support systems but also as educational aids. In a previous study, a machine learning model was developed to classify tympanic membrane images into normal, otitis media with effusion (OME), chronic otitis media (COM), and cholesteatoma. The model achieved a diagnostic accuracy of 97.2% on a representative dataset. Importantly, when ten otolaryngology residents were asked to diagnose 71 tympanic membrane images with and without AI assistance, their diagnostic accuracy increased from an average of 82.9% to 90.0% after consulting the model’s output. The individual improvement ranged from 1.4% to 18.3%, demonstrating the model’s capacity to augment clinical judgment and reduce diagnostic error [3]. These findings suggest that AI-based otoscopic analysis not only achieves expert-level performance but may also serve as a valuable educational and clinical support tool, especially in settings where access to specialized expertise is limited.

The diagnostic performance of the proposed multi-class classification model was comparable to or exceeded that of previous studies. A study developed an AI-based classifier using tympanic membrane video data to detect acute otitis media (AOM) and reported a sensitivity of 93.8% and specificity of 93.5% for binary classification (AOM vs. no AOM), utilizing deep residual-recurrent neural networks [21]. In contrast, the present study addressed a more complex four-class classification problem (normal, AOM, OME, and COM), achieving an overall accuracy of 88.7%, with F1-scores of 0.78 for AOM, 0.87 for OME, and 0.92 for COM. In 2022, a Korean study demonstrated high diagnostic performance for a three-class classification model (OME, COM, and normal) using the EfficientNet-B4 architecture, reporting a Dice similarity coefficient (DSC) of 95.19%, with OME and COM achieving 93.8% and 96.1%, respectively. While that study included secondary categories and used segmentation-assisted training, the present model uniquely incorporated a complete preprocessing pipeline including image quality filtering, tympanic membrane segmentation, and laterality classification [4]. Another investigation evaluated convolutional neural network (CNN) architectures for the detection of cholesteatoma, reporting classification accuracies ranging from 83.8% to 98.5% depending on the model and task type [8]. In contrast to these single-disease-focused approaches, this study utilized a unified pipeline to classify multiple subtypes of otitis media using still images, offering broader diagnostic utility for general clinical use.

This study has several limitations. First, the dataset was collected retrospectively from a single tertiary care center, which may limit the generalizability of the model to broader clinical settings, especially in primary care or community-based environments. Second, although the model demonstrated high performance in classifying still images, it was not evaluated using real-time video otoscopy or in live clinical scenarios. Third, all ground-truth labels were assigned by a single senior otologist. Future studies should incorporate consensus labeling by multiple experts to reduce subjectivity and further validate the robustness of the model. Fourth, the otoscopic images used in this study were acquired at a moderate resolution (640 × 480 pixels), which is lower than that of contemporary high-definition digital otoscopes. This may have limited the model’s ability to capture very fine anatomical details. Future studies incorporating prospective multi-center data, real-time integration into clinical workflows, and consensus labeling may further enhance the reliability and clinical applicability of AI-based otoscopic diagnostic tools.

## 5. Conclusions

The present study demonstrated the potential of a deep learning-based diagnostic pipeline to support the automated classification of tympanic membrane images across multiple types of otitis media. This structured, multi-step approach to otoscopic diagnosis provides a practical solution to improve diagnostic consistency and accessibility. These findings underscore the potential for the continued development and clinical integration of AI-assisted tools in middle ear disease diagnosis.

## Figures and Tables

**Table 1:** Classification performance by diagnostic category.

	Precision	Recall	F1-Score	Support
Normal	0.86	0.91	0.89	1052
AOM	0.79	0.77	0.78	232
OME	0.90	0.84	0.87	700
COM	0.93	0.92	0.92	980
Accuracy	-	-	0.89	2964
Macro Average	0.87	0.86	0.86	2964
Weighted Average	0.89	0.89	0.89	2964

**Table 2:** The confusion matrix for the diagnostic results of the machine learning networks.

		Prediction			
		Normal	AOM	OME	COM
**Diagnosis**	**Normal**	960	10	40	42
	**AOM**	14	178	18	22
	**OME**	78	28	586	8
	**COM**	58	9	9	904

**Table 3:** Classification performance across K-fold cross-validation: accuracy, sensitivity, specificity, and AUC for each fold.

	K-Fold				
	1	2	3	4	5
**Accuracy**	87.52%	88.36%	87.35%	88.53%	91.55%
**Sensitivity**	92.95%	92.17%	91.10%	91.36%	93.19%
**Specificity**	87.14%	91.90%	91.94%	89.10%	96.19%
**AUC**	0.96	0.97	0.97	0.97	0.98

## Data Availability

The datasets used and/or analyzed during the present study are available from the corresponding author on reasonable request.
